# Inhibition of Publication and Its Results

**Published:** 1877-10

**Authors:** 


*Inhibition of Publication and its Results.*—It is stated as a curious result of a late attempt at suppressing a book in England, that its sale rose from 500 copies before the indictment was lodged, to 125,000 copies since!

